# Epidemiological and genomic analyses of human isolates of *Streptococcus suis* between 2005 and 2021 in Shenzhen, China

**DOI:** 10.3389/fmicb.2023.1118056

**Published:** 2023-03-27

**Authors:** Liyin Ji, Zhigao Chen, Fan Li, Qinghua Hu, Liangcai Xu, Xiangke Duan, Hanguang Wu, Shiqin Xu, Qiongcheng Chen, Shuang Wu, Shuxiang Qiu, Huiqun Lu, Min Jiang, Rui Cai, Yaqun Qiu, Yinghui Li, Xiaolu Shi

**Affiliations:** ^1^School of Public Health, Shanxi Medical University, Taiyuan, China; ^2^Shenzhen Center for Disease Control and Prevention, Shenzhen, China; ^3^Shenzhen Institute of Quality and Safety Inspection and Research, Shenzhen, China; ^4^Futian District Center for Disease Control and Prevention, Shenzhen, China; ^5^School of Public Health, University of South China, Hengyang, China

**Keywords:** *Streptococcus suis*, human, epidemiology, SNP-based phylogeny, pathogenicity island, antimicrobial resistance

## Abstract

*Streptococcus suis* (*S. suis*) is an important food-borne zoonotic pathogen that causes swine streptococcosis, which threatens human health and brings economic loss to the swine industry. Three-quarters of human *S. suis* infections are caused by serotype 2. A retrospective analysis of human *S. suis* cases in Shenzhen, a megacity in China, with high pork consumption, between 2005 and 2021 was conducted to understand its genomic epidemiology, pathogen virulence, and drug resistance characteristics. The epidemiological investigation showed that human cases of *S. suis* in Shenzhen were mainly associated with people who had been in close contact with raw pork or other swine products. Whole-genome sequence analysis showed that 33 human isolates in Shenzhen were dominated by serotype 2 (75.76%), followed by serotype 14 (24.24%), and the most prevalent sequence types (STs) were ST7 (48.48%) and ST1 (39.40%). ST242 (9.09%) and ST25 (3.03%), which were rarely reported, were also found. Phylogenetic analysis showed that the Shenzhen human isolates had close genetic relatedness to isolates from Guangxi (China), Sichuan (China), and Vietnam. We found a new 82 KB pathogenicity island (PAI) in the serotype 2 isolate that may play a role in sepsis. Similarly, a serotype 14 isolate, containing 78 KB PAI, was isolated from a patient presenting with streptococcal toxic shock syndrome (STSLS) who subsequently died. Multi-drug resistance (MDR) was high in human isolates of *S. suis* from Shenzhen. Most human isolates were resistant to tetracycline, streptomycin, erythromycin, and clindamycin, and 13 isolates had intermediate resistance to penicillin. In conclusion, swine importation from Guangxi, Sichuan, and Vietnam should be more closely monitored, and the use of antibiotics limited to reduce the potential for antimicrobial resistance (AMR).

## 1. Introduction

Shenzhen is a megacity in China, with a population of approximately 20 million, and has a high consumption of pork due to its high nutritional value. There are no swine farms in Shenzhen, and live swine are imported from nearby provinces and cities and exported, for example, to Hong Kong and Macao. *Streptococcus suis* (*S. suis*) is a significant zoonotic pathogen that is localized in the upper respiratory tract of healthy swine (Goyette-Desjardins et al., 2014). In humans, *S. suis* can cause meningitis, septicemia, endocarditis, pneumonia, arthritis, and streptococcal toxic shock syndrome (STSLS), with pathogen transmission through broken skin contact with swine, pork, and pork-related derivatives or consumption of raw or undercooked pork products (Kerdsin et al., 2022). In severe cases, *S. suis* infection can lead to death (Goyette-Desjardins et al., 2014). China has had three outbreaks of *S. suis* serotype 2 in Jiangsu (1998), Sichuan (2005), and Guangxi (2016), which caused multiple infections and deaths, and brought serious economic loss to the localities (Tang et al., 2006; Huang et al., 2019b).

To date, 29 authentic *S. suis* serotypes (1–19, 21, 23–25, 27–31, and 1/2; Okura et al., 2016), serotype Chz (Pan et al., 2015), and 26 novel capsular polysaccharide loci (NCL) have been described (Zheng et al., 2015; Qiu et al., 2016; Zheng et al., 2017; Huang et al., 2019a), depending on the antigenicity of the capsular polysaccharide. Of human pathogenic isolates, 74.7% are *Streptococcus suis* serotype 2 (*S. suis* 2), which is the most common and virulent isolate across geographic regions (Goyette-Desjardins et al., 2014). In addition, *Streptococcus suis* serotype 14 (*S. suis* 14) has been isolated several times from human isolates (Huang et al., 2019b; Kerdsin et al., 2020; Thu et al., 2021). Multi-locus sequence typing (MLST) is commonly used to distinguish between pathogen isolate types. PubMLST shows that 1,962 different sequence types (STs) of *S. suis* have been identified worldwide, and new STs are constantly being discovered. Among them, ST7 is mostly endemic to China (Goyette-Desjardins et al., 2014). The 89 KB pathogenicity island (PAI) was discovered in the Chinese *S. suis* 2-ST7 isolates, 98HAH12, SC84, and 05ZYH33 (Ye et al., 2006; Chen et al., 2007) and contained several genomic components associated with pathogenicity. Such genomic components include three groups of ABC transport systems controlling substance transport, a two-component signal transduction system (TCS), three type IV secretory system (T4SS) component genes, a toxin-antitoxin system, and a Tn*916* transposon (Wu et al., 2011). These genomic components played an important role in STSLS and drove the *S. suis* outbreak in Jiangsu (1998) and Sichuan (2005) of China (Chen et al., 2007).

Both human and veterinary prevention of *S. suis* infection and disease treatment depends on the effective use of antibiotics, which are equivalent or belong to the same class (Yongkiettrakul et al., 2019). In recent years, however, antibiotic overuse on a global scale has caused bacteria to evolve antimicrobial resistance (AMR), and multi-drug resistance (MDR) is gradually increasing (Aradanas et al., 2021). There is a similar concern that *S. suis* may act as a streptococci community (pathogenic and non-pathogenic) reservoir for antibiotic resistance genes (ARGs; Palmieri et al., 2011). High-level AMR to tetracyclines, macrolides, and lincosamides has been reported in both human and swine isolates of *S. suis* (Gurung et al., 2015; Ichikawa et al., 2020; Aradanas et al., 2021). The ARGs, *tet*(O) and *erm*(B), are the most common genes associated with AMR (Princivalli et al., 2009; Chen et al., 2013b), and consequently, beta-lactamase and fluoroquinolone antibiotics are used to treat *S. suis* infections in both swine and human (Aradanas et al., 2021). However, non-penicillin and non-levofloxacin susceptible isolates of *S. suis* have also been reported worldwide (Varela et al., 2013; Bamphensin et al., 2021; Dechêne-Tempier et al., 2021). As treatment for *S. suis* infection in both humans and swine is dependent on antibiotics, monitoring antibiotic sensitivity will help to optimize antibiotic therapy.

A total of 43 human cases of *S. suis* infection were reported in the Shenzhen region over a 17-year time period^1^. The epidemiological characteristics of the infections remain undescribed, and the genetic relatedness among humans of Shenzhen and global *S. suis* isolates remains unclear. To understand the possible relationship between the human *S. suis* isolates of Shenzhen and the global isolates from a phylogenetic perspective, we collected and preserved isolates from 33 of the 43 cases from 2005 to 2021 and performed a genomic analysis. Antibiotic susceptibility testing was also performed to investigate the potential resistance of *S. suis* isolates in Shenzhen and provide a basis for clinical treatment of human *S. suis* infection.

## 2. Materials and methods

### 2.1. Sample collection, isolation, and identification

A total of 43 human cases of *S. suis* were reported in Shenzhen of China from 2005 to 2021, as determined using PulseNet China. Cerebrospinal fluid or blood was taken from each of the 43 patients at the time of hospitalization, and isolates from 33 of the 43 cases were obtained from sentinel hospitals (Supplementary Table S1) and sent to Shenzhen Center for Disease Control and Prevention. Clinical information and patient exposure history 1 week prior to disease onset were also collected from China Information System for Disease Control and Prevention. Samples (*n* = 100) of clinically healthy swine tonsils with quarantine certificates from Abattoir 2 in Shenzhen were collected in December 2021. Each swine tonsil tissue was aseptically cut into small pieces, then homogenized in 1 ml of PBS using a tissue homogenizer (MagNA Lyser Instrument, United States), delineated on Columbia blood agar medium (Detgerm Microbiogical Science Ltd., Guangzhou, China), and incubated at 37°C for 18–24 h in a 5% CO_2_ incubator. From each sample, 1–2 small gray–white colonies with varying degrees of hemolysis were selected and inoculated on Columbia blood agar medium and incubated at 37°C for 18 to 24 h. A single colony was placed in an EP tube (1.5Ml) filled with 300 μl of pure water and bathed at 100°C for 15 min to extract nucleic acid. Using the *Streptococcus suis* detection kit (real-time PCR method; MABSKY, Shenzhen, China) for the conserved gene *gdh* of *S. suis*, individual suspected colonies were identified with a Ct value of ≤ 36 and a significant curve growth, which could be judged as positive isolates. There were 38 positively identified *S. suis* isolates from the swine samples. Including both the human and swine samples, a total of 71 *S. suis* isolates were included in the study.

### 2.2. Genomic DNA preparation, whole-genome sequencing, assembly, and annotation

Genomic DNA was obtained from all 71 *S. suis* isolates using the Ezup Column Bacteria Genomic DNA Purification Kit (Sangon Biotech, Shanghai, China), after resuscitation and passaging using the Columbia Blood Plate. Pair-end libraries with a mean insert size of 350 bp were prepared using the NEB Ultra DNA Library Preparation Kit (NEB, Massachusetts, United States). Whole-genome sequencing was conducted using Illumina NovaSeq PE150 (Novogene Co. Ltd., Beijing, China); the average read length was 150 bp, yielding an average of 1 GB of clean reads per isolate. Short-read sequencing data for the Shenzhen isolates have been stored in the NCBI Sequence Read Archive under accession number PRJNA894855. In addition, 429 genome sequences of *S. suis* 2 and *S. suis* 14 isolates were downloaded from the NCBI SRA (375) and NCBI Assembly (54) for further analysis. Data information on 429 genome sequences is provided in Supplementary Table S2.

A total of 344 genome sequences downloaded from the NCBI SRA, and 71 pair-ended genome sequences in this study were filtered using the software trimmomatic v0.39 (Bolger et al., 2014), and then, Kraken2^2^ was used to identify *S. suis*. SPAdes v1.1.0 (Bankevich et al., 2012) was used to perform *de-novo* genome assembly. Quast v5.0.2 (Gurevich et al., 2013) was used to check the quality of all 500 draft sequences, and Prokka 1.14.6 (Seemann, 2014) was used to perform gene annotation.

### 2.3. Serotyping and MLST

*Streptococcus suis* serotyping pipeline^3^ was used for *S. suis* serotyping, used to identify the traditional 29 serotypes and differentiate between serotypes 2 and 1/2, and between serotypes 1 and 14. For serotypes other than the 29 traditional serotypes, a cps database was created based on published articles, containing serotype Chz (Pan et al., 2015) and NCL1-26-specific genes (Zheng et al., 2015, 2017; Qiu et al., 2016; Huang et al., 2019a). SRST2 (Inouye et al., 2014) was used to perform serotyping in comparison with the created database. The genome sequences of the isolates were submitted to the *S. suis* MLST database^4^ for genotype determination, and then, clonal complex groups (CCs) were identified using goeBURST^5^.

### 2.4. Phylogenetic tree building

**Figure 1:**
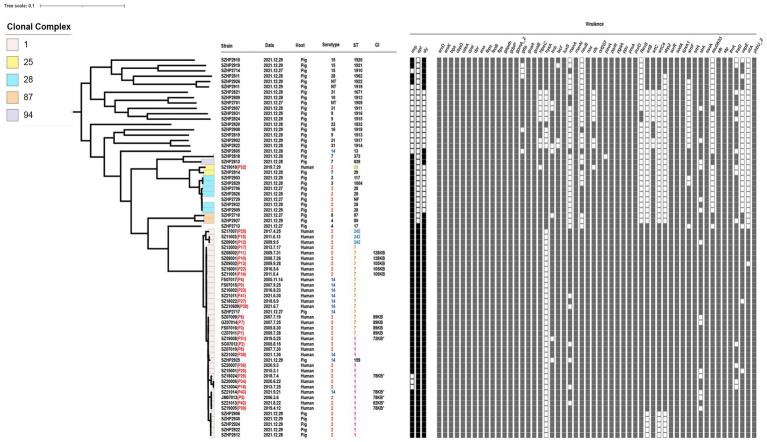
Information and virulence genes of 71 *Streptococcus suis* isolates in Shenzhen. The red font after the strain number is the patient number. Data mean the onset time of human isolates and the isolation time of swine isolates. Serotype, ST, clone complex, and host of 71 isolates are described. “*” indicates that it is derived from 89 KB PAI. Squares are filled with color when the corresponding virulence gene is present.

**Figure 2:**
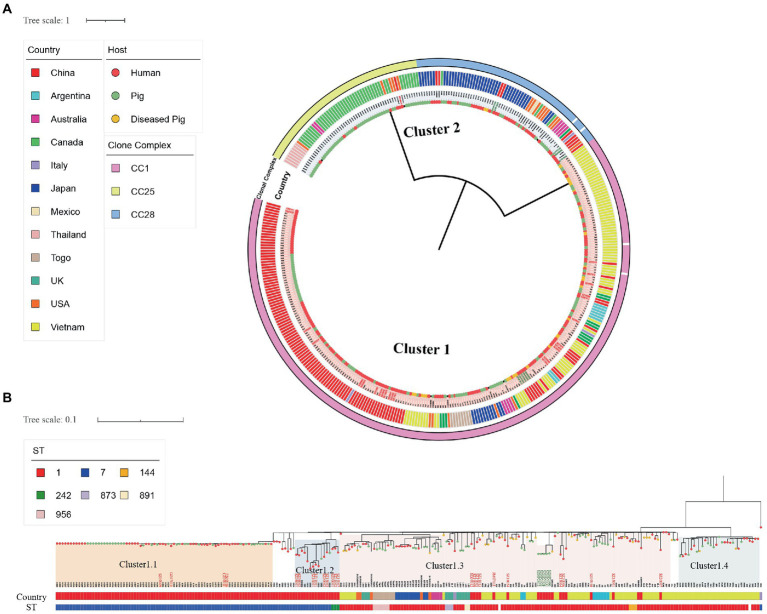
Single-nucleotide polymorphism-based global *S. suis* 2 Ml tree. **(A)** SNP-based global *S. suis* 2 Ml tree includes 25 human isolates (red font) and 10 swine isolates (green font) from Shenzhen, and 345  *S. suis* isolates downloaded from the NCBI database. The different colored circles on each branch represent different host sources, and the circles represent clonal complex, country, or cluster as indicated on the figure. **(B)** SNP-based global *S. suis* 2 Cluster1 ML tree includes 24 human isolates (red font) and five swine isolates (green font) from Shenzhen, and 224  *S. suis* isolates downloaded from the NCBI database. The country and host legends of the isolates are the same as on **(A)**. ST or cluster as indicated on the figure.

**Figure 3:**
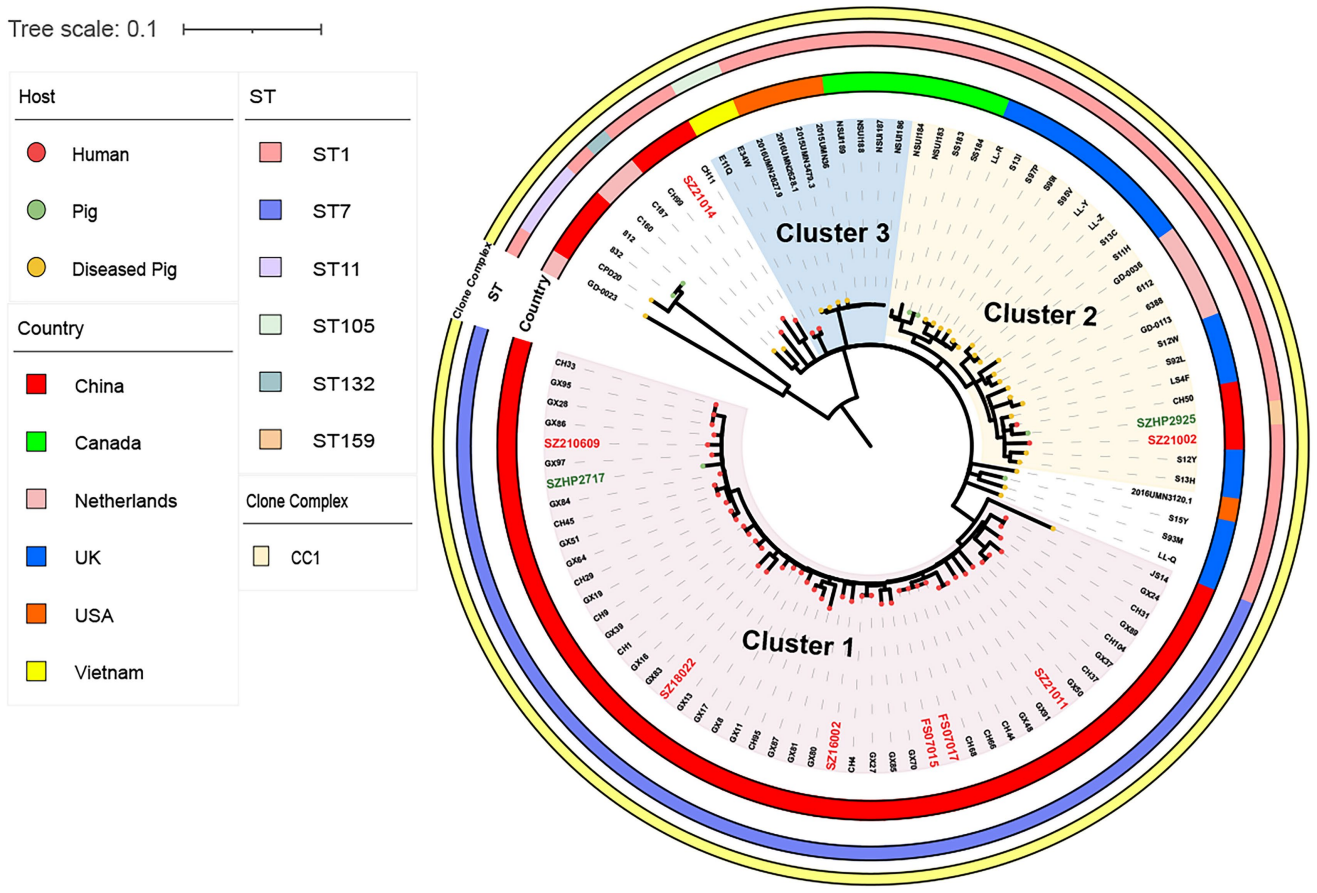
Single-nucleotide polymorphism (SNP)-based global *S. suis* 14 Ml tree includes eight human isolates (red font) and two swine isolates from Shenzhen (green font), and 86  *S. suis* isolates downloaded from the NCBI database. The different colored circles on each branch represent different host sources, the circles represent clonal complex, ST, or country, as indicated on the figure.

Core-genome (regions present in > 99% of isolates) single-nucleotide polymorphisms (core-SNPs) were identified using Snippy Pipeline v4.6.0^6^. SC84 (accession number: NC_012924.1) was used as the reference sequence for the Shenzhen isolate sequences (Figure 1) and *S. suis* 2 sequences (Figure 2), and JS14 (accession number: NC_017618.1) was used as the reference sequence for *S. suis* 14 sequences (Figure 3). Gubbins v2.4.1 (Croucher et al., 2015) was used to remove recombination, and SNP distance matrices were obtained for isolates using snp-dist v0.6.3^7^, and then, non-repetitive core-SNPs were used to construct the three maximum-likelihood (ML) trees using FastTree v2.1.10 (Price et al., 2010), with the auto-detected best-fitting substitution model. iTOL^8^ was used for modification and presentation (Figures 1–3).

**Figure 4:**
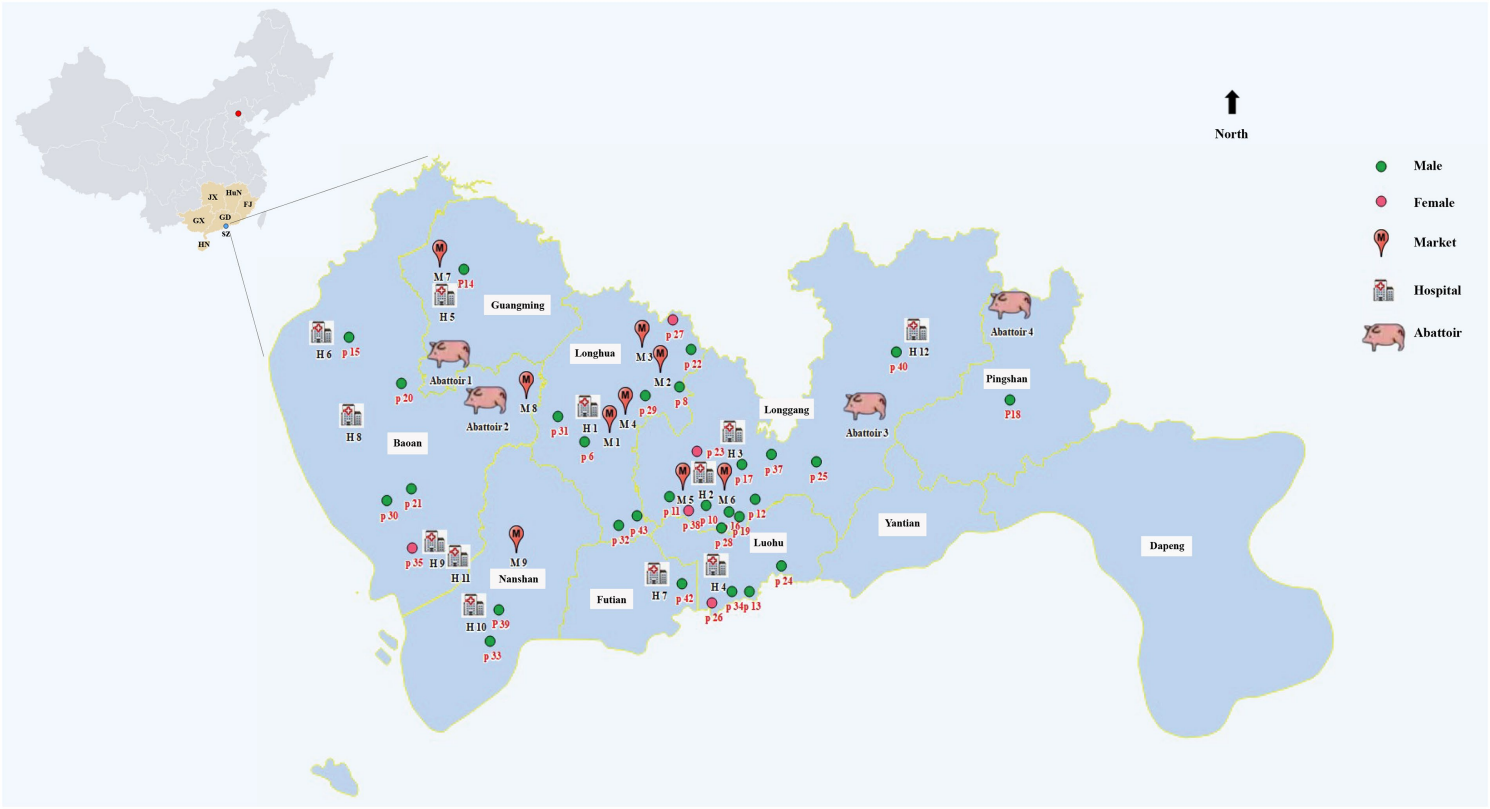
Map of domestic sources of pork and the distribution of human cases of *S. suis* in Shenzhen, China. The orange area of the map of China (left) represents the source of pork imports to Shenzhen. The letters indicate the provinces of China: GX = Guangxi; HuN = Hunan; JX = Jiangxi; FJ = Fujian; GD = Guangdong; HN = Hainan; SZ = Shenzhen. The blue area (right) is an expanded representation of the Shenzhen region. The circles in the figure represent the distribution of cases in each district of Shenzhen.

### 2.5. Identification of virulence genes, plasmids, and prediction of gene islands

To identify virulence genes in the isolates, a database containing 84 virulence genes was created based on previously published data (Dong et al., 2015; Cucco et al., 2022), using Abricate^9^ and in combination with the VFDB online database^10^. PlasmidFinder v2.1 (Carattoli et al., 2014) was used to detect plasmids. A database of *S. suis* gene island (GI) was also constructed, containing 89 K PAI present in epidemic isolate 05ZYH33 (Li et al., 2011), the 105 K GI found in the diseased swine isolate SC070731(Wu et al., 2014), and the 128 k GI found in CMGETZ080501 (Huang et al., 2016). Blast v2.5.0 (Camacho et al., 2009), with a cutoff value of ≥ 70% coverage and ≥ 80% identity was used to determine the presence of GI. The results were presented using Easyfig^11^.

### 2.6. Antibiotic susceptibility testing and identification of ARGs

The antimicrobial susceptibility of isolates to 17 antibiotics across 10 classes (Aminoglycosides, Amphenicols, Sulfanilamides, Lincosamides, Macrolides, Tetracyclines, Oxazolidinones, Fluoroquinolones, β-lactams, and Glycopeptides; Supplementary Table S3) was determined using the minimal inhibitory concentration (MIC) method. The broth microdilution method (FOSUN DIAGNOSTICS, Shanghai, China) was used to determine the antibiotic sensitivity of the *S. suis* isolates. Chloramphenicol (CPL), compound sulfamethoxazole (SMZco), clindamycin (DA), erythromycin (ERY), tetracycline (TC), linezolid (LZD), levofloxacin (LEV), moxifloxacin (MXF), penicillin (PG), cefepime (FEP), cefotaxime (CTX), meropenem (MEM), amoxicillin (AML), vancomycin (VA), and teicoplanin (TEC) were tested. The E-test method (Liofilchem, Italy) was used to determine the antibiotic sensitivity of the *S. suis* isolates to streptomycin (SM) and ceftriaxone (CRO). *Streptococcus pneumoniae* ATCC 49619 was used as a quality control isolate. Breakpoints for sensitive, intermediate, and resistant types were defined using the Clinical and Laboratory Standards Institute (CLSI) document M100 (31st edition; Humphries et al., 2021) when available, otherwise, EUCAST (v 13.0) clinical breakpoint cutoff values^12^ were used. The reference breakpoint of SM was determined based on the high-level resistance values for SM in Streptococcus suis reported by Marie J. (Marie et al., 2002). In the absence of clinical breakpoints from CLSI or EUCAST, the values obtained were displayed. Resfinder 4.1 (Bortolaia et al., 2020) was used to identify ARGs.

### 2.7. Statistical analysis

Mean ± standard deviation (SD) and frequency were used to describe the characteristics of *S. suis* cases and isolates. Categorical variables were compared using the chi-square and Fisher’s exact tests. A two-sample *t*-test was performed to access the differences between the two means. Wilcoxon rank-sum test was used when the continuous variables were not normally distributed. A consistency check was used to verify the consistency of the two methods. All analyses were performed using SPSS 27.0.1. *p*-values of less than 0.05 were considered statistically significant.

## 3. Results

### 3.1. Epidemiological and clinical features

Among the 43 cases of human *S. suis* reported in Shenzhen from 2005 to 2021, 34 cases were scattered across nine Shenzhen districts (Figure 4; Supplementary Table S1). There was a high prevalence of *S. suis* cases across the four districts of Longgang (27.91%), Longhua (18.60%), Baoan (11.63%), and Luohu (9.30%), though no obvious epidemiological association between them. Nine cases were imported from other cities in Guangdong of China, though seven of these cases had missing information. The remaining 36 cases had a minimum age of 22 years and a maximum age of 84 years, with a mean age of 50.14 (±12.96) years. A total of 31 (86.11%) of the *S. suis*-infected patients were male. Almost four-fifths of patients were cooks (41.67%), butchers (25.00%), and freelancers (16.67%) with close contact with raw pork or swine products. *S. suis* infections presented throughout the year, with a higher incidence in the hot and humid climate between April and September in Shenzhen.

Of the 36 patients with clinical information, we did not collect the clinical type of five of these patients, and 21 patients were diagnosed with meningitis (58.33%), four patients with sepsis (11.11%), and one patient with arthritis (2.78%). Three patients had meningitis complicated by sepsis (8.33%), and two patients had STSLS and died (5.56%). The mortality rate among 36 cases was 5.56%. Among the exposure history of the 36 patients, 28 patients had a history of raw pork exposure, of which 12 patients had wounds on their hands and did not use any personal protection when in contact with raw pork, and two patients had tasted raw pork or eaten pork from diseased swine. Exposure history data were lost from six patients. In addition, prior to the disease onset, 2 of the 30 patients had a history of exposure to chicken and duck meat only and had not been exposed to or consumed pork or related products. Patients’ epidemiological and clinical data are provided in Supplementary Table S1.

### 3.2. *Streptococcus suis* serotyping, ST, and CCs identification in Shenzhen

Of the total 71 *S. suis* isolates in Shenzhen (Figure 1), three-quarters of the human isolates were *S. suis* 2 (*n* = 25, 75.76%), and the remaining isolates were *S. suis* 14 (*n* = 8, 24.24%). The swine isolates (*n* = 38) carried a wide range of serotypes, though *S. suis* 2 was the most common (*n* = 10, 26.32%). Other swine isolate serotypes included serotypes 2, 3, 4, 7, 8, 9, 10, 14, 15, 16, 23, 28, and 31, and non-typeable ones.

Multi-locus sequence typing (MLST) identified four STs in human isolates (*n* = 33), which were present in differing prevalence. ST7 was the most prevalent (48.48%), followed by ST1 (39.40%), then ST242 (9.09%), and finally ST25 (3.03%). A total of 22 STs were identified in the swine isolates (*n* = 38), among which ST1 (13.16%) and ST28 (10.53%) were the most prevalent. Eight new STs were identified in the swine isolates; ST1909, ST1911, ST1912, ST1913, ST1914, ST1917, ST1918, and ST1962, and a further isolate could not be genotyped. eBURST analysis showed that ST1, ST7, ST159, and ST242 belonged to CC1; ST25 and ST29 belonged to CC25; ST28, ST117, and ST1004 belonged to CC28; ST87 and ST89 belonged to CC87; ST373 and ST839 belonged to CC94; and other STs appeared in the single case form.

### 3.3. Phylogenetic analysis of Shenzhen human isolates with global *Streptococcus suis* isolates

To study the relationship between Shenzhen human *S. suis* 2 and global *S. suis* 2 isolates, we compared 25 human isolates of *S. suis* 2 in Shenzhen with 10 swine isolates of *S. suis* 2 from this study, and 345 global *S. suis* 2 isolates. Then, we constructed an *S. suis* 2 Ml tree of 380 isolates based on 7,370 SNPs in the core genome. The ML tree was divided into two clusters (Figure 2A), with Cluster1 containing CC1 isolates and Cluster2 containing CC25 and CC28 isolates. Shenzhen human isolates were mainly concentrated on Cluster1, which was further divided into four subgroups (Figure 2B). Within Cluster1, four ST7 (16.00%) Shenzhen human isolates isolated from 2005 to 2007 distributed to the Cluster1.1 subgroup were closely related to the Sichuan (China) isolates of 2005 (0 ≤ snp ≤ 4). Six ST7 (24.00%) and three ST242 (12.00%) Shenzhen human isolates isolated from 2008 to 2017 were distributed to the Cluster1.2 subgroup and were genetically close to the diseased swine isolate HN08324 of Henan (China; 8 ≤ snp ≤ 23) and Guangxi (China) human isolate GX9 (10 ≤ snp ≤ 23). In addition, 11 ST1 (44.00%) Shenzhen human isolates isolated from 2005 to 2021 in the Cluster1.3 subgroup were more closely related to the Vietnamese human isolates B34E and S8V(6 ≤ snp ≤ 19). Furthermore, a Shenzhen human isolate SZ19010 (4.00%), which belongs to ST25, is located in Cluster2 and was genetically distant (snp ≥ 5,622) from the remaining 24 (96.00%) Shenzhen human isolates of *S. suis* 2. SZ19010 had a closer genetic relationship with isolates from the United States, Canada, Thailand, and Australia (6 ≤ snp ≤ 68) and was most closely related to United States swine isolate NSUI012 and diseased swine isolates, 2014UMN2148.7 and 2016UMN1440.1 (snp = 6).

In the same way, we compared eight Shenzhen human and two swine *S. suis* 14 isolates from this study with 86 global *S. suis* 14 isolates. A *S. suis*14 ML tree was constructed with 96 isolates based on 1,688 SNPs in the core genome and divided into three clusters (Figure 3). Six ST7 (75.00%) Shenzhen human isolates isolated from 2005–2021 in Cluster1 were closely related to the Guangxi (China) human isolates GX8, GX17, and GX84 (17 ≤ snp ≤ 31), while the Shenzhen ST1 human isolate SZ21002 located in Cluster2 was closely related to the UK-diseased swine isolate LS4F (snp = 35) and Guangxi (China) human isolate CH29 (snp = 39), and the other ST1 human isolate SZ21014 isolated in 2021was closely related to Guangxi (China) human isolate CH11 (snp = 50). The information on genome sequences data is provided in Supplementary Table S2.

### 3.4. Virulence genes and plasmids of Shenzhen isolates and 89 K PAI detection

A total of 57 virulence genes were detected in the genomes of 71 Shenzhen isolates (Figure 1). By the Wilcoxon rank-sum test, the CC1 carried more virulence genes (55.92 ± 0.17) than other CCs (45.53 ± 0.69; *p* < 0.001). Therefore, CC1 was defined as a cluster of highly virulent clones. It consisted of *S. suis* 2 and *S. suis* 14 isolates, containing 32 human isolates (96.97%) and seven swine isolates (18.42%). All isolates in CC1 showed *mrp + epf + sly +*, except for SZ13004, SZ18024, and SZ20006, which showed *mrp- epf + sly +*. For non-CC1 swine isolates, although some were *S. suis* 2 or *S. suis* 14, they only carried one of the three virulence genes. In addition, a swine serotype 4-ST17 isolate presented with *mrp + epf + sly +*. The only Shenzhen human *S. suis* 2-ST25 isolate SZ19010 belonging to CC25 showed *mrp + epf–sly–*.

**Figure 5:**
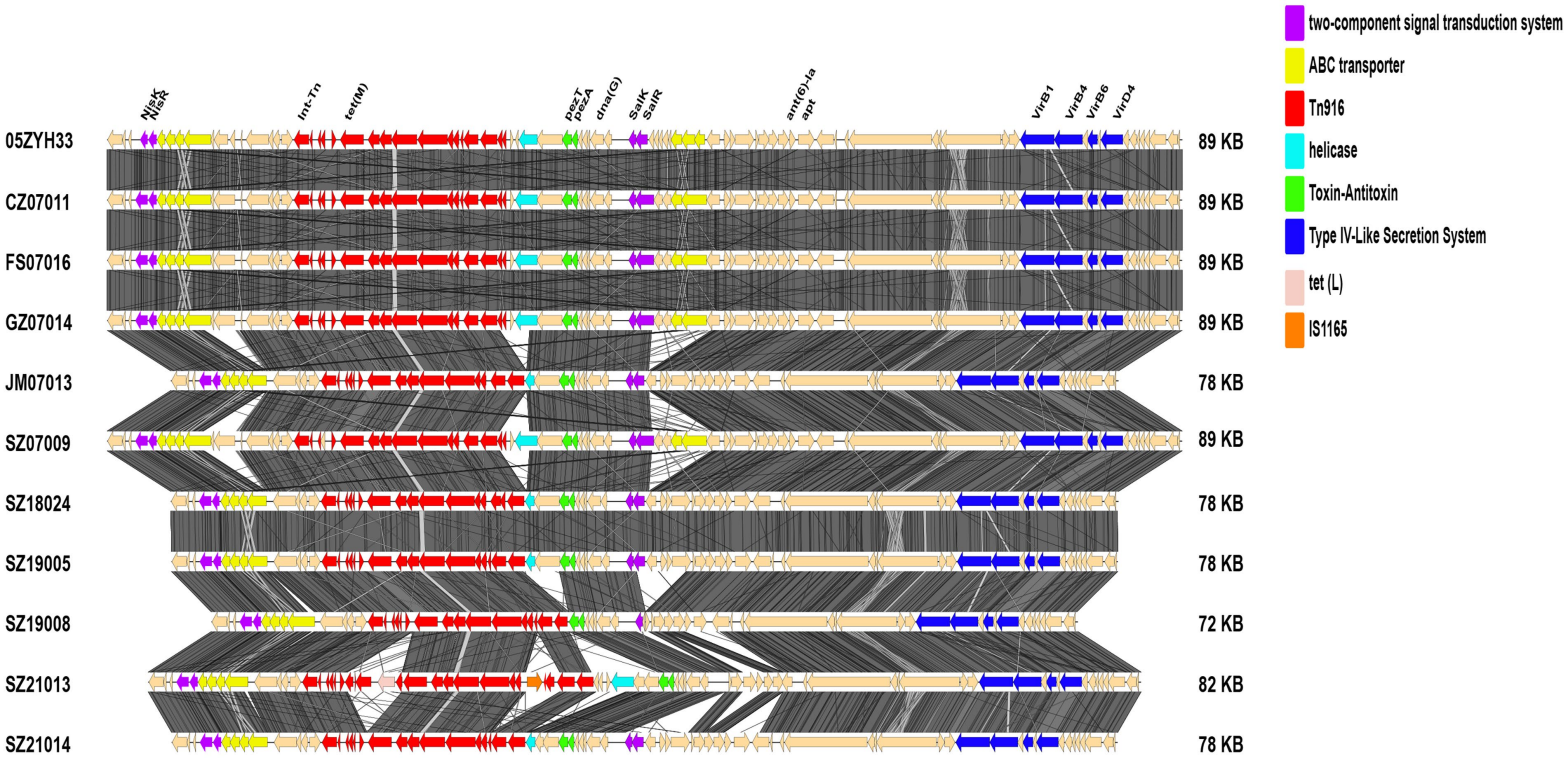
Structural diagram of the 89 KB PAI and its variants found in the Shenzhen isolates.

In the CC1 human isolates, we found that the four *S. suis* 2-ST7 isolates that were genetically closely related to the Sichuan isolates (2005), had a complete 89 KB PAI. We also found 78 KB, 72 KB, and 82 KB PAI, which were derived from 89 KB PAI. Four *S. suis* 2-ST1 human isolates were found to have 78 KB PAI and retained most of the key factors associated with virulence, with only one group of ABC transporter proteins missing. We found a 72 KB PAI in a human isolate SZ19008, which lost the *SalR* gene in the TCS, in addition to a set of ABC transporter proteins. Clinical information indicated that the patient had STSLS and died. We identified an 82 KB PAI in a human isolate SZ21013, which had lost one of the TCS *SalK/SalR*, in comparison with the prevalent isolate 05ZYH33. In addition, many fragments in the Tn*916* transposon were lost, and TC resistance-related efflux pump gene *tet*(L) and the ISL3 family transposase IS1165 were inserted. The 78 KB PAI was also found in the *S. suis* 14 isolate SZ21014 from a patient presenting with STSLS. As the 78 KB PAI found in the *S. suis* 2 isolates, the one in *S. suis* 14 carried all the 89 KB PAIs except for a group of ABC transporter proteins (Figure 5). No plasmids were detected in the 71 Shenzhen isolates.

### 3.5. Antimicrobial resistance phenotype testing and gene detection

Drug susceptibility testing (Supplementary Table S3) showed that 33 human isolates of *S. suis* exhibited high levels of resistance to the antibiotics: TC (*n* = 32, 96.97%), SM (*n* = 21, 63.64%), ERY (*n* = 18, 54.55%), and DA (*n* = 18, 54.54%). In addition, 39.39% (*n* = 13) of the isolates demonstrated intermediate resistance to PG (0.25 ≤ MIC≤1 μg/ml). A total of 38 swine isolates also exhibited high levels of resistance to the antibiotics TC (*n* = 38, 100%), DA (*n* = 35, 92.11%), and ERY (*n* = 33, 86.84%). In addition, swine isolates also had resistance to SM (*n* = 9, 23.68%), LEV (*n* = 4, 10.53%), and CPL (*n* = 1, 2.63%). Swine isolates demonstrated intermediate resistance to PG (*n* = 5, 13.16%), CPL (*n* = 1, 2.63%), and LEV (*n* = 1, 2.63%). By chi-square test, the resistance rate of the swine isolates to ERY and DA was higher than that of the human isolates (*p* < 0.05).

A total of 11 different resistance patterns were observed in this study (described in Table 1). The MDR rate of 33 human *S. suis* isolates was 72.73% (*n* = 24), and the main resistance patterns were Ami-Lin-Mac-Tet. Meanwhile, the MDR rate of 38 swine *S. suis* isolates reached 89.47% (*n* = 34), and the main resistance pattern was Lin-Mac-Tet.

**Table 1:** Phenotypic antimicrobial resistance (AMR) profiles of *Streptococcus suis* isolated from Shenzhen, China.

Phenotype	Human (n = 33)		Swine (n = 38)	
	n	%	n	%
Ami-Lin-Mac-Tet	10	30.30	6	15.79
Ami-Tet-Bla	6	18.18	0	0.00
Ami-Lin-Mac-Tet-Bla	2	6.06	1	2.63
Lin-Mac-Tet	4	12.12	21	55.26
Lin-Mac-Tet-Bla	2	6.06	0	0.00
Ami-Amp-Lin-Mac-Tet-Flq-Bla	0	0.00	1	2.63
Ami-Amp-Lin-Mac-Tet	0	0.00	1	2.63
Lin-Tet-Flq	0	0.00	1	2.63
Lin-Mac-Tet-Flq	0	0.00	1	2.63
Ami-Lin-Mac-Tet-Flq-Bla	0	0.00	1	2.63
Ami-Lin-Mac-Tet-Flq	0	0.00	1	2.63
Sum	24	72.73	34	89.47

Ami, Aminoglycosides; Lin, Lincosamides; Mac, Macrolides; Tet, Tetracyclines; Bla, β-lactams; Amp, Amphenicols; Flq, Fluoroquinolones.

**Figure 6:**
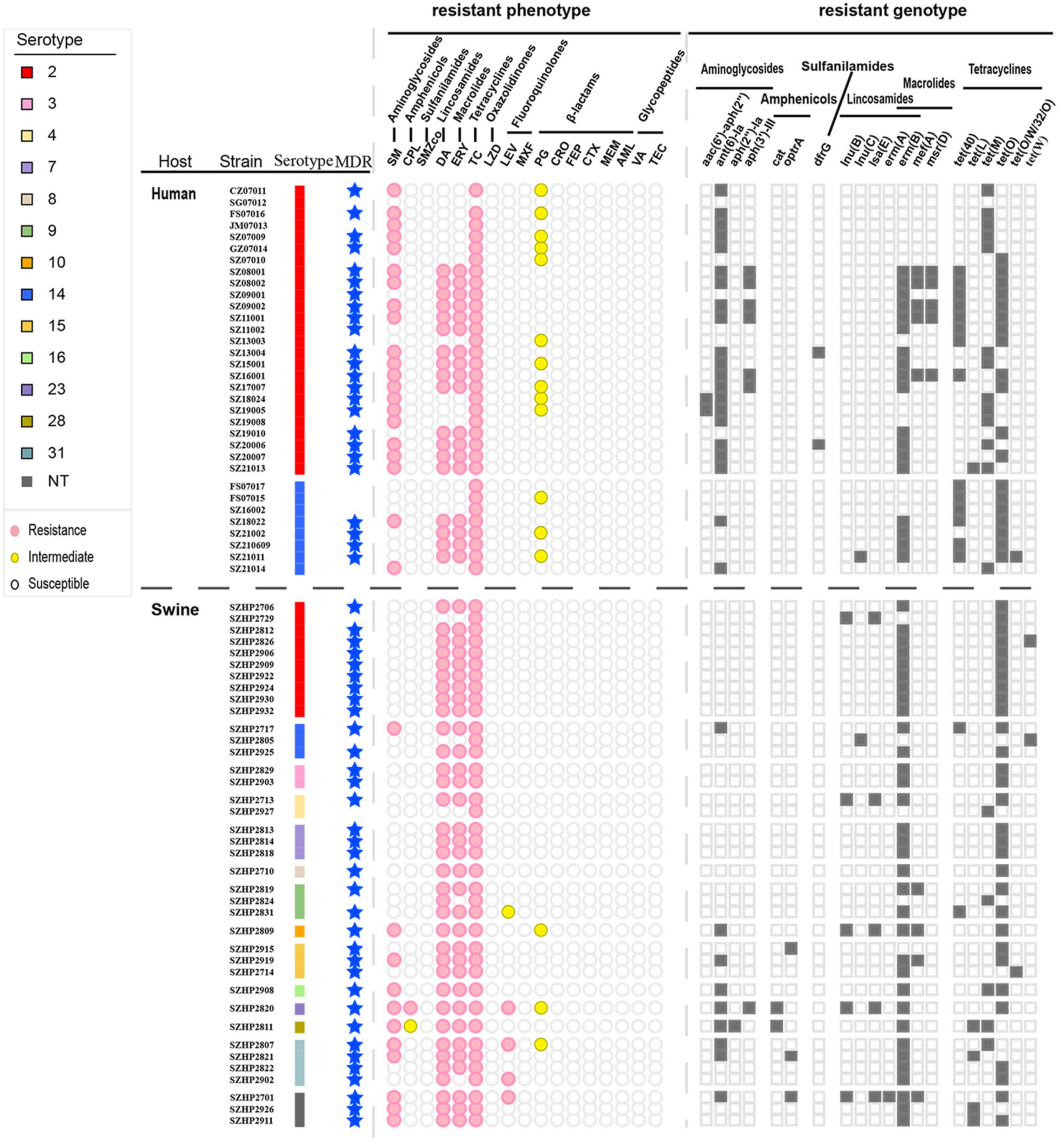
Different serotypes of Shenzhen *S. suis* isolates with resistance phenotype and resistance genotype distribution. MDR = multiple drug resistance. Circles or squares are filled with color when the corresponding resistance phenotype or resistance genotype is present.

A total of 20 ARGs were detected in the 71 isolates, including aminoglycosides (*n* = 4), amphenicols (*n* = 2), sulfonamides (*n* = 1), lincosamides (*n* = 3), macrolides (*n* = 4), and tetracyclines (*n* = 6; Figure 6). Among human isolates, *ant*(*6*)*-Ia* of aminoglycosides was the most common (63.64%), followed by *tet*(O) of tetracyclines (57.58%) and *erm*(B) of macrolides-lincosamides (54.55%). Among the swine isolates, macrolides-lincosamides *erm*(B) was the most common (86.84%), followed by tetracyclines *tet*(O) (78.95%) and aminoglycosides *ant*(*6*)*-Ia* (23.68%). Further comparison of human and swine *S. suis* 2 and *S. suis* 14 isolates by Fisher’s exact test revealed that *ant*(*6*)*-Ia, tet*(40), *tet*(M), and *tet*(O) were distributed differently among hosts and serotypes (*p* ≤ 0.05). Results showed that in human isolates, *ant*(*6*)*-Ia* was more predominant in *S. suis* 2 isolates and *tet*(40) was more predominant in *S. suis* 14 isolates. When comparing human and swine *S. suis* 2 isolates, *ant*(*6*)*-Ia* and *tet*(M) were more frequently present in human isolates, while *tet*(O) was found more frequently in swine isolates (Supplementary Table S4). By analyzing the consistency of antimicrobial resistance and phenotypes among 71 isolates in Shenzhen, it was found that there was a good agreement between resistance genotypes and phenotypes for SM, DA, ERY, TC, LZD, PG, CRO, FEP, CTX, MEM, AML, and VA (Kappa values = 1, *p* < 0.001).

## 4. Discussion and conclusion

*Streptococcus suis S. suis* infection can be fatal to humans and is transmitted from swine and related products. Based on phylogenetic results, we found that Shenzhen human isolates had close genetic relatedness to isolates from Guangxi (China), Sichuan (China), and Vietnam. ML Cluster1.1 was formed by the *S. suis* 2-ST7 Sichuan 2005 outbreak isolates (Li et al., 2011). Shenzhen human isolates in this cluster were endemic only for a short time in 2005–2007, potentially due to the quarantine and mass destruction of swine in response to the large-scale *S. suis* outbreak. In addition, from 2005–2021, the Shenzhen human *S. suis* isolates were mainly closely related to isolates from Guangxi and Vietnam. Most of the pork in Shenzhen comes from Guangxi, Guangdong, and other neighboring Chinese provinces (Zongyun and Guojian, 2019). Guangxi is close to Vietnam, and a private swine trade exists between them (Kedkovid et al., 2020). Huang et al. (2019b) demonstrated a close relationship between Guangxi and Vietnamese isolates of *S. suis* from a phylogenetic perspective. Meanwhile, the phenomenon of Vietnamese swine smuggling into Guangdong is also quite prominent (Magazine, 2014), which may enable the introduction of Vietnamese pathogen isolates into Guangxi and Guangdong and then into Shenzhen. Unfortunately, the Guangxi and Vietnam isolates that could be included in this study were human isolates, and the relationship between swine and humans could not be found, suggesting that we should strengthen the monitoring of the *S. suis* of swine.

Four human ST242 isolates have been found in human cases of meningitis, one in Guangxi (Jiang et al., 2020), and three in this study (distributed in Figure 2B Cluster1.2). There is no conclusive evidence of direct human-to-human *S. suis* transmission (Gottschalk et al., 2010). We only found one swine ST242 isolate (PubMLST), though there is no evidence that human ST242 isolates came from swine. ST242 has a certain pathogenicity, and the detection of ST242 in swine and human isolates could be strengthened for comparative analyses. In addition, the reported *S. suis* 2-ST25 isolates were mainly isolated from the United States, Australia, and Thailand (Kerdsin et al., 2018; Segura et al., 2020) and were relatively rare in China, with only one ST25 isolate recorded from a diseased swine (Zhu et al., 2013) and one from a patient in Hong Kong (Luey et al., 2007) have been reported. In this study, we found an ST25 isolate SZ19010 which was located in *S. suis* 2 Ml Cluster2 had close genetic relatedness to isolates from the USA. The import volume of frozen meat to Shenzhen ranks third in China and is mainly from the EU, the United Kingdom ^13^, Brazil, and the USA (Lowell et al., 2018; Zu et al., 2020). It suggests that the import of frozen meat also has the risk of human infection. In addition, we should sample the swine that are transported into Shenzhen to study the presence proportion and pathogenic ability of ST25 in Shenzhen swine.

Dong et al. (2021) divided China *S. suis* isolates into three lineages. Both lineagesIand III are ST7 isolates, with lineage I isolate containing 89 KB PAI. Lineage III represents a novel ST7, lacking 89 KB PAI but containing 128 KB GI, and lineage II is ST1 isolate, containing 78 KB PAI. Similar results were found in the human isolates from Shenzhen. Among the Shenzhen human isolates of *S. suis* 2, the ST7 isolates at Cluster1.2 were missing 89 KB PAI but had inserted 105 KB or 128 KB GI. In comparison, the ST7 isolates at Cluster1.1, carrying *ant*(*6*)*-Ia*, *aph*(*3′*)*-III*, *erm*(B), *mef*(A), *msr*(D), *tet*(O), and *tet*(40) ARGs, demonstrated MDR to the antibiotics SM, DA, ERY, and TC. In addition, apart from ST1 isolates located in Cluster1.3, isolates contained 78 KB PAI with all the key factors associated with virulence in 89 KB PAI. Conversely, we found 72 KB and 82 KB PAI in *S. suis* 2 Cluster1.3, which were distinct from 89 KB PAI due to missing all or part of the *SalK/SalR*. *Tet*(L) and ISL3 family transposase IS1165 were found in 82 KB PAI. *Tet*(L) is usually carried in *Streptococcus* on small transmissible plasmids and was detected in the Tn*916* transposon of the Vietnamese human isolate BM407 (Holden et al., 2009). It is not present in isolates representative of the 2005 outbreak in China, such as SC84 and 05ZYH33 (Holden et al., 2009). While IS1165 was initially reported to be present in *Leuconostoc mesenteroides* subsp*. Cremoris* (Johansen and Kibenich, 1992), in this study, IS1165 was found for the first time in *S. suis* PAI. It is, therefore, necessary to further investigate the structure and function of *S. suis* 82 KB PAI fragments that may be responsible for changing pathogenicity and drug resistance. Further confirmation of relative pathogenicity is needed. 78 KB PAI was generally prevalent in *S. suis* 2 isolates (Dong et al., 2021), and we also found it in human isolates of *S. suis* 14. Therefore, we need to further study whether the pathogenicity of 78 KB PAI changes in different serotypes.

In this study, we found there was a good agreement between resistance genotypes and phenotypes for SM, DA, ERY, TC, LZD, PG, CRO, FEP, CTX, MEM, AML, and VA. This finding suggests that ARGs can therefore be used to predict resistant *S. suis* phenotypes. Currently, clinical treatment of *S. suis* infection is mainly with the antibiotics PG and CRO (Segura et al., 2020). We found that 13 isolates had intermediate resistance to PG. The emergence of isolates with intermediate resistance to PG suggests that the antibiotic treatment strategy should be promptly adjusted, and antibiotics used sparingly to avoid antibiotic resistance.

We found that carriage of *S. suis* was common in clinically healthy swine, and there was a diversity of serotypes and genotypes, including serotypes 2, 4, 7, 9, 14, 16, and 31, which are closely related to human infection (Liang et al., 2021; Wang et al., 2021). High virulence isolates were always found on the same branch (Chen et al., 2013a), and we found eight swine isolates clustered with 32 human isolates in Shenzhen, possessing the three key virulence factors *mrp*, *sly*, and *epf*. This indicates that healthy swine may act as reservoirs for *S. suis* that can then be transmitted to humans.

It is widely reported that the risk of *S. suis* infection is higher in people who come in close contact with swine and pork-related products, particularly with wound exposure (Feng et al., 2014; Lu et al., 2021). Most studies on *S. suis* have therefore focused on swine; however, we found two cases of *S. suis* infection resulting from exposure to, or consumption of chicken. In a study of Vietnamese chicken flocks, (Nhung et al., 2020) suggested that chickens may be a source of *S. suis* infection in humans and swine. These findings suggest that the carriage rate and genotype of *S. suis* should be monitored in poultry and swine.

To prevent human *S. suis* infection, meat should be bought with a quarantine certificate. In addition, personal and environmental hygiene should be maintained when handling raw meat and contact with diseased or dead swine and poultry, their excrement, and body fluids should be avoided. Occupational personnel working in direct contact with swine and poultry should wear protective gloves and clothing as well as rubber shoes. Workers with skin injuries should avoid contacting with swine, poultry, and their meat, and follow strict hygiene protocols, including covering wounds, wearing protective, and frequent hand washing. Cross-contamination of raw and cooked meat should also be avoided during food processing.

In summary, effective monitoring of imported swine and poultry from Guangxi and other provinces should be implemented to prevent the occurrence and spread of human *S. suis* disease in Shenzhen. At the same time, international cooperation should be strengthened to control the smuggling of live swine along with the China-Vietnam border. In addition, health education for relevant occupational groups should be increased to improve self-protection awareness, which can effectively avoid the occurrence of swine streptococcal disease. Finally, to reduce the burden of bacterial AMR, the rational use of antibiotics is urgently needed.

## Data availability statement

The data presented in the study are deposited in the NCBI repository, accession number PRJNA894855.

## Ethics statement

The studies involving human participants were reviewed and approved by the Ethics Committee of the Shenzhen Center for Disease Control and Prevention (QS2022110079). Written informed consent from the participants’ legal guardians/next of kin was not required to participate in this study, in accordance with the national legislation and the institutional requirements.

## Author contributions

LJ and XS conceived and designed the study. ZC collected case-related data. FL collected the swine samples. LJ, QH, LX, XD, HW, SX, QC, SW, SQ, HL, MJ, RC, YQ, and YL performed the experiments and data analysis. LJ drafted the manuscript. XS revised the manuscript. LJ, ZC, and FL contributed equally to this work. All authors contributed to the article and approved the submitted version.

## Funding

This research was supported by the National Natural Science Foundation of China (No. 81773436), the Key Scientific and Technological Project of Shenzhen Science and Technology Innovation Committee (KCXFZ202002011006190), Shenzhen Key Medical Discipline Construction Fund (SZXK064), Non-profit Central Research Institute Fund of Chinese Academy of Medical Sciences (2020-PT330-006), and the Sanming Project of Medicine in Shenzhen (NO. SZSM201811071).

## Conflict of interest

The authors declare that the research was conducted in the absence of any commercial or financial relationships that could be construed as a potential conflict of interest.

## Publisher’s note

All claims expressed in this article are solely those of the authors and do not necessarily represent those of their affiliated organizations, or those of the publisher, the editors and the reviewers. Any product that may be evaluated in this article, or claim that may be made by its manufacturer, is not guaranteed or endorsed by the publisher.

## Footnotes

^1^ https://10.249.6.18:8881/cdc/login

^2^ https://ccb.jhu.edu/software/kraken2/

^3^ https://github.com/streplab/SsuisSerotyping_pipeline

^4^ https://pubmlst.org/ssuis/

^5^ https://online.phyloviz.net

^6^ https://github.com/tseemann/snippy

^7^ https://github.com/tseemann/snp-dists

^8^ https://itol.embl.de/

^9^ https://github.com/tseemann/abricate

^10^ http://www.mgc.ac.cn/VFs/

^11^ https://github.com/mjsull/Easyfig

^12^ www.EUCAST.org

^13^ https://agriculture.Ec.Europa.Eu/farming/animal-products/pork_en
